# Facial display and blushing: Means of visual communication in blue-and-yellow macaws (*Ara Ararauna*)?

**DOI:** 10.1371/journal.pone.0201762

**Published:** 2018-08-22

**Authors:** Aline Bertin, Arielle Beraud, Léa Lansade, Marie-Claire Blache, Amandine Diot, Baptiste Mulot, Cécile Arnould

**Affiliations:** 1 PRC, CNRS, IFCE, INRA, Université de Tours, Nouzilly, France; 2 ZooParc de Beauval & Beauval Nature, Saint-Aignan, France; University of California San Diego, UNITED STATES

## Abstract

Mainly recognized for their cognitive performance, the visual communication system and, particularly, the potential function of facial displays in parrots remain thus far unexplored. Here, we provide the first descriptive study of facial display use in captive blue-and-yellow macaws. We observed the feather position (sleeked or ruffled) on the crown, nape and cheek at the group level during the macaws’ daily routine and individually while interacting with a familiar animal caretaker. In the latter context, blushing was also assessed on the bare skin of the cheek. Group level observations showed that crown, nape and cheek feathers ruffling was more frequent in activities requiring no locomotion than in activities requiring locomotion. With the animal caretaker, crown ruffling was significantly more frequent when the caretaker was actively engaging with the parrot than during a control phase with no mutual interaction. In addition, a significantly higher proportion of naïve observers judged blushing as being present on photographs taken during the mutual interaction phase than during the control phase. We thus showed significant variations in facial displays and bare skin colour based on the birds’ social context and activity. Our results broaden the scope for further studies to determine whether parrots’ faces provide visual social signals.

## Introduction

In addition to their primary functions in flight and thermoregulation, bird plumage also provides visual signals within and between species. Body displays, sometimes associated with conspicuous plumage colouring, are particularly well described in the contexts of mate acquisition and agonistic interactions [1,2]. In many avian species with sexual plumage-colour dimorphism, complex visual signals are thought to have evolved through females’ sexual selection since variation in the visualized plumage properties may convey signals regarding mate quality or fighting ability [3–6].

Although the Psittaciformes (parrots, cockatoos and lorikeets; called parrots hereafter for simplicity) provide some of the most impressive examples of animal colouration, the function of plumage in visual communication has been far less investigated in this order (for a review [7]. Two reasons may explain this: little or no sexual dimorphism in plumage colours (but see [8]) and the colour-production system unique to the animal kingdom. Parrots do not express carotenoid-based colouration (in which variation may convey information on mate quality) but a colouration based on a unique pigment class called psittacofulvins, which are not related to diet [9]. The implication of plumage in acquiring mates has received recent interest but remains poorly understood in parrots [10]. Complex visual threat displays are described in the parrot genus, *Trichoglossus* [11], and anecdotally in nesting blue-and-yellow macaws [12]. Moreover, to our knowledge, body feather use in social contexts unrelated to sexual selection or aggressive interactions remains overlooked in parrots.

Our study aimed to deepen the understanding of visual communication in parrots. We focused our attention on the head/face of non-breeding, captive blue-and-yellow macaws. Despite the absence of facial muscles [13], birds dispose of a feather-bearing integument with contractile properties [14]. In addition, as described in detail by [15], birds’ heads are composed of several sections where feathers can be erected or sleeked independently of one another.

Mammalian faces have been well described to convey public information regarding their intention to engage in specific activities or emotions [16]. In primates, literature on facial expression relates the importance of these discrete visual signals in group cohesion and functioning (e.g. [17,18]). Although parrots are highly social with primate-like cognitive capacities [19], the potential use of facial micro-signals remains overlooked. Some authors [15,20,21] have reported that discrete movements of feathers on birds’ heads may convey affective information such as mood (e.g., play mood) or emotion.

Blue-and-yellow macaws are appropriate for such a study since their head is composed of mobile, coloured feathers on top of their crown and nape, mobile black lines of feathers on their cheeks and white bare skin. The bare skin is also of interest since blushing can be observed (rapid colour variation from white to red) but has not been studied in macaws. Much less research has been conducted on rapid changes in avian skin colouration. Blushing is reported in 12 different avian orders based on blood flow in vascularized tissues [22], but the function of this response remains unknown. In Lappet-faced vultures (*Aegypius tracheliotos*), facial blushing has been hypothesized to play a role in agonistic interactions [23]. Rapid changes in bare skin colour have also been anecdotally reported in the crested caracara (*Caracara cheriway*) when excited or stressed [24,25].

Here we first aimed to describe crown, nape and cheek feather positions during different behavioural activities of the birds. To that end, we observed a group of five macaws during their daily routine in their aviaries. We hypothesised that there would be variation in the expression of feather ruffling dependent on the activity of the birds.

In a second part of the study, we used the human-animal relationship context to investigate whether variations in the feather position and skin colour (blushing) could be observed in situations potentially differing in emotional valence. Captive parrots bond strongly with humans (e.g. [26]) and are sensitive to mutual attention. The presence of joint avian-human attention can, for example, improve learning allospecific speech in grey parrots (*Psittacus erithacus*) [27]. Therefore, to observe possible variations in the feather position and skin colour, we observed these parameters while the birds were mutually interacting with a familiar caretaker (positive valence) or in the absence of mutual attention (control phase, less positive valence). We hypothesized that if the feather ruffling frequency and blushing occurrence varied significantly between the two phases, that these parameters may indicate differences in inner subjective appraisal of the situation. We aimed to distinguish subtle visual displays that may help to better understand the parrots’ communication system. This applied knowledge may be important for species that are commonly kept as pets as well as for conservation programs where welfare indicators in captive conditions, such as indicators of positive emotions, remain to be determined.

## Materials and methods

### Birds and housing conditions

We observed five hand-reared blue-and-yellow macaws, not exposed to the public, at the Zooparc de Beauval Saint Aignan (41110, France). All birds are part of a free-flying show. The birds had been trained daily (i.e., handled daily) since weaning and were thus in close contact with humans, especially their caretakers. All the birds had fully adult-like plumage but were not yet sexually mature and did not express sexual or defensive behaviours. The five birds were housed in two adjacent aviaries of similar sizes with an indoor area (250 cm x 520 cm x 260 cm) freely connected to an outdoor area (250 cm x 850 cm x 260 cm). Three of the macaws were housed in aviary 1 (Table 1). The other two were housed in aviary 2 with a red macaw (*Ara macao*) and a yellow-crested cockatoo (*Cacatua sulphurea citrinocristata*). The aviaries were equipped with several tree branches, perches and ropes. Enrichment was provided daily (cardboard and journal paper). Parrots were fed daily with fresh fruits and vegetables, germinated seeds (wheat, corn, sunflower, rice, and oat), millet seeds, oyster shells, and a commercial mix for exotic birds.

**Table 1:** Age, sex and location of the 5 captive macaws used for the study.

Individuals	Age (years)	Sex	Aviary
Aruba	3	female	1
Antonio	3	male	1
Imotep	1	unknown	1
Gédéon	4	female	2
Petry	1	unknown	2

### Behaviour and feather postures

#### Video-recording and image analysis

To determine during which activity feathers were ruffled on the crown, cheek and nape, we used a focal sampling method with a hand held camera recorder (Sony HDRP PJ410) capturing 24 images per second. We followed one focal bird’s behaviour for twenty minutes, and then successively followed another of the five birds. Each twenty-minute session was repeated (on different days) in a random order until we obtained two hours of recording per bird. As birds were trained for free flight daily in the morning, we observed the birds in the afternoon between 2 and 5 PM. The experimenter was familiar to them and moved around the aviary when necessary with the minimum of disturbance possible.

**Fig 1:**
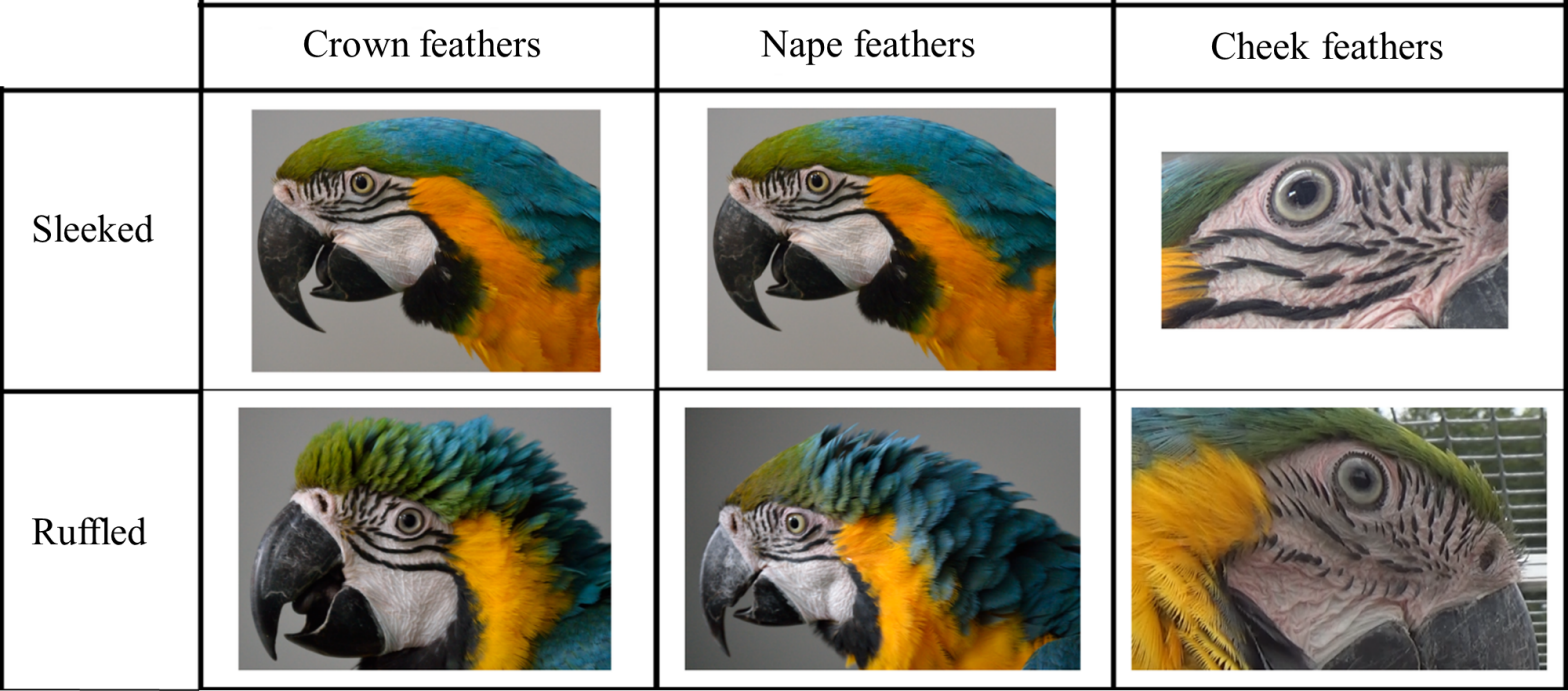
Repertoire of head feather displays. Photographic representation of the position of crown, nape and cheek feathers. (All photographs taken by A. Beraud).

We used a scan sampling method to analyse the videos. Every 5 s, the experimenter recorded the bird’s behaviour following the behavioural repertoire presented in Table 2 and simultaneously, the feather position following the repertoire presented in (Fig 1). From preliminary observations, we determined three areas where feathers can move independently from one another: the crown (composed of green feathers), the nape (composed of blue, yellow and black feathers) and the cheek (Fig 1). For each area, we determined two feather positions: sleeked or ruffled. When the cheek feathers are sleeked, continuous black lines can be observed, and when they are ruffled, the lines appear discontinuous. For the crown and nape, when sleeked, one cannot distinguish individual feathers or see their tips. When ruffled, individual feathers and their tips can be distinguished. When some parameters were unobservable, the scan was deleted. We obtained a mean total number of 1327.8 ± 37.2 scans per individual (minimum: 1198; maximum: 1407). The same experimenter conducted all observations. We assessed the observer reliability by rescoring 20 min of video per bird. The percentage of intra-observer accordance was 90.53%.

**Table 2:** Behavioural repertoire used to record each bird’s activity while in its social group.

Behaviour	Description
Locomotion	walking, flying, climbing
Alimentation	the bird is ground-foraging and eating, drinking
Chewing	the bird is chewing pieces of wood or enrichments
Maintenance	preening, scratching, stretching
Social interactions	allopreening, perched in body contact without or with interactions (touching the conspecific with the beak or the feet)
Resting	the bird is immobile	

The observations were conducted in April and May 2017 with temperatures ranging from 15°C to 22°C.

### Feather postures and blushing when interacting with the animal caretaker

#### Protocol

We aimed to determine whether variations in feather position and skin colour could be observed when interacting with the most familiar caretaker. The caretaker has trained birds for free-flight daily for months and was thus very familiar to the birds (during free-flight training the birds were taught to come to hand when called after unrestricted outdoor flight).

For each session, each bird was taken by hand at random by the caretaker and transported a few meters away to an empty aviary (250 cm x 520 cm x 260 cm) equipped with a familiar perch and a camera recorder mounted on a tripod (Sony HDRP PJ410). All birds were used to being handled for daily training and weighing. Each bird was then placed on the perch in front of the animal caretaker. Each session was composed of two 2-minute phases.

Phase 1: Mutual interaction. During this phase, the animal caretaker actively interacted with the bird by looking at and talking to them (no scratch or massage).

Phase 2: Control. In a control context, we observed each bird in the presence of the animal caretaker but with no mutual interaction. During this phase, the animal caretaker remained at the same distance but turned her back to the bird. We considered this phase as a less positive valence for the birds than phase 1. This phase also allowed observing whether the birds expressed “seeking behaviour” towards the animal caretaker (the bird tried to grasp the caretaker’s clothes with its beak or claws, bent on its side and looked at the caretaker, or jumped on the caretaker). We hypothesized that if the mutual interaction was positive for the birds, they would more positively seek attention from the animal caretaker to restore the active interaction during the control phase than during phase 1. At the end, the bird was transported back to its aviary.

We repeated the entire procedure (phases 1 and 2) ten times (10 sessions) per bird. All sessions were conducted in the afternoon on two successive weeks with only one session per day. We did not counterbalance the order of the two phases because in prior trials, the birds were unwilling to stay on the perch for the first two minutes if not actively engaged.

#### Image analysis

Each phase (phase 1: mutual interaction, phase 2: control) was video recorded with a Sony HDRR PJ410 capturing 24 images per second. Positive seeking behaviours were assessed on videos for each bird and phase.

With the same protocol as described in the first part of the study, we used a scan sampling method every 5 s on the video to assess the feather position (ruffled or sleeked) based on the repertoire (Fig 1). We obtained a mean ± SE total number of 213.60 ± 4.91 scans per individual (minimum: 205; maximum: 227) during the control period and a mean total number of 211.20 ± 9.70 scans per individual (minimum: 175; maximum: 228) during the mutual interaction.

**Fig 2:**
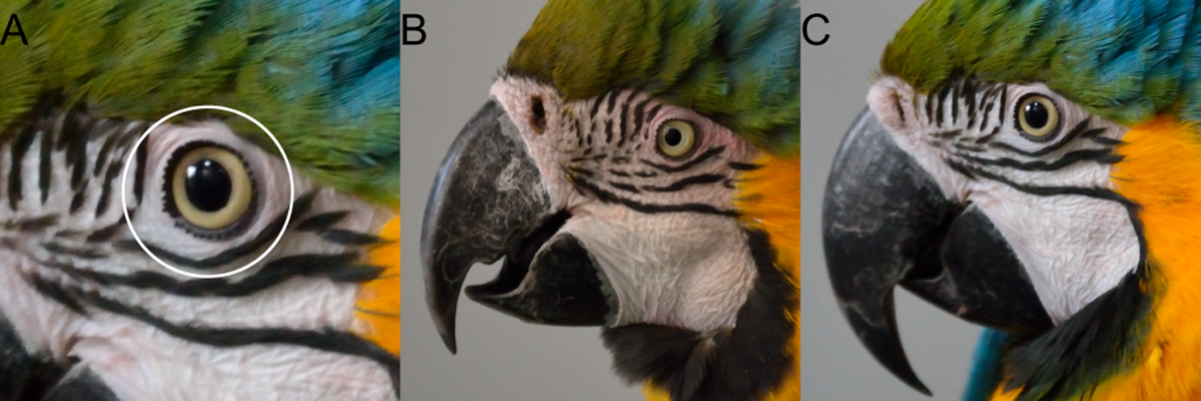
Examples of photographs. A) Target zone used to estimate the presence or absence of blushing by a panel of 4 naïve observers. B) Photograph where all observers judged blushing to be present in the target zone. C) Photograph where no observers judged blushing to be present in the target zone. (All photographs taken by A. Beraud).

We also took one close-up photograph of the bird’s left profile at the end of each phase (20 photographs per bird) with a Nikon D3100. To assess the presence or absence of blushing on the bare white cheek skin, 100 images were taken with 20 images per bird (1 per phase x 10 sessions). Three images were discarded due to blurring (all with the caretaker’s back to the bird). A panel of four naïve observers visually assessed the presence or absence of blushing on the ring of bare skin around the eye (Fig 2). This zone was chosen from preliminary observations when we observed that blushing was not diffused homogeneously throughout the entire bare skin patch surface. This area around the eye blushed the most frequently. The percentage of inter-observers accordance was 88.15%.

### Statistics

For behaviour and feather posturing observations in each behavioural category, we calculated the mean proportion of ruffled feather scans per bird. We used the function aovp of the lmPerm package in R 3.4.2 to run permutation tests with activity type as a fixed factor and individual as a random factor. Due to the small sample size, we reduced the number of pairwise comparisons and defined two main activity types: 1) activities requiring locomotion including the behavioural categories “locomotion” and “alimentation” or 2) activities requiring no-locomotion including the behavioural categories: “chewing”, “maintenance”, “social interactions” and “resting” (defined in Table 2). To determine whether feather posturing was concomitant or not between the three anatomical regions, for each of the following pairs: crown-nape, crown-cheek and cheek-crown, we compared the proportions of scans where feather position was similar or different between the two anatomical regions to a random distribution (50% chance) with Chi2 tests.

For feather postures and blushing when interacting with the animal caretaker we used the function aovp of the lmPerm package in R 3.4.2, to run permutation tests with the phase as a fixed factor, the individual as a random factor and the session as a random factor nested within individuals. For seeking behaviours, the tests were applied on the number of behaviours observed per phase, per individual and per session. For crown and nape feathers, the permutation tests were applied on the proportions of scans where ruffling was observed per phase, per individual and per session. Cheek feathers ruffling was not observed. For blushing, the permutation tests were applied on the proportions of observers classifying the photographs as “with blushing” per phase, per individual and per session. All tests were two-tailed with significance considered at *P* < 0.05. The data are represented as boxplots with medians and interquartile distribution ranges.

### Ethical note

The Zooparc de Beauval (41110, Saint Aignan) kindly provided access to their birds. Only video-recorded observations were conducted. Behavioural observations are not considered as experimentations and are beyond the scope for ethical consideration regarding French and European animal experimentation regulations. Therefore, the Ethics Committee for Animal Experimentation of Val de Loire, CEEA Vdl considered that ethical approval was not required for this study. These animals are free-flight trained and have a confident relationship with their caretakers.

## Results

### Behaviour and feather posture

**Fig 3:**
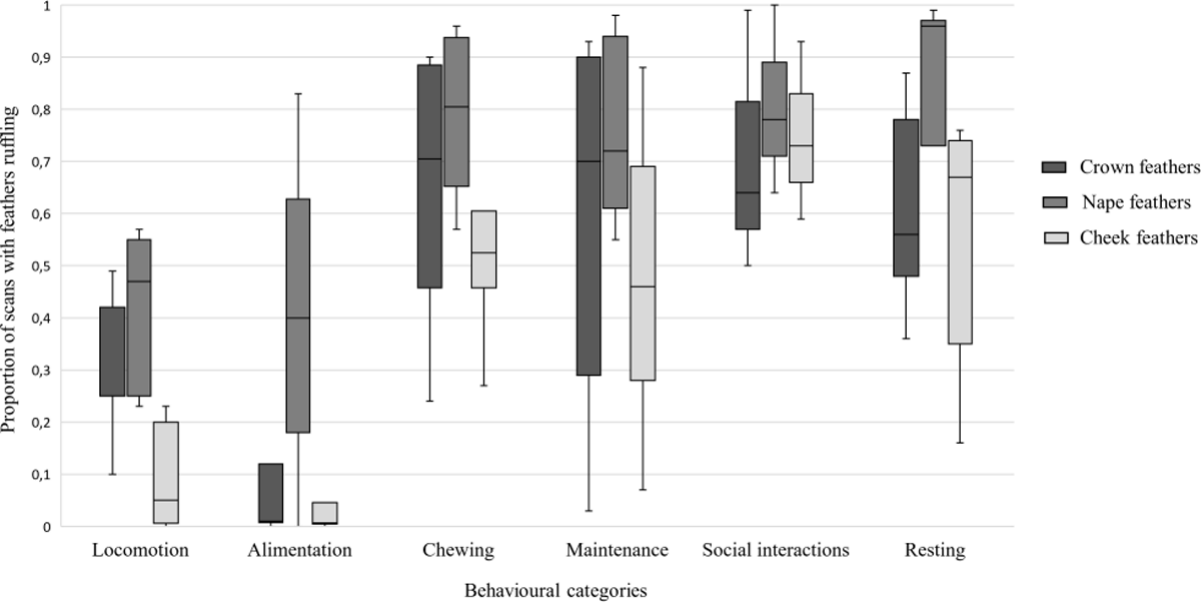
Median and interquartile distribution ranges of the proportions of scans where feathers ruffling was observed. The proportions of scans are represented for the behavioural categories: locomotion (total mean ± SE number of scans = 96 ± 24), alimentation (73 ± 28 scans), chewing (149 ± 48 scans), maintenance (153 ± 39 scans), social interactions (108 ± 59 scans), resting (746 ± 103 scans).

The proportions of scans where feathers ruffling was observed were significantly lower during activities requiring locomotion (locomotion, alimentation) than during activities requiring no locomotion (chewing, maintenance, social interactions, resting) for the crown (*df* = 1; Mean Square (*MS*) = 1.06; *P* < 0.01), the nape (*df* = 1; *MS* = 0.97; *P* < 0.01) and the cheek (*df* = 1; *MS* = 1.33; *P* < 0.01) (Fig 3). We found no significant effect of the random factor individual on the fixed effect activity type for the crown (*df* = 4; *MS* = 0.19; *P* = 1), the nape (*df* = 4; *MS* = 0.02; *P =* 1) and the cheek (*df* = 4; *MS* = 0.04; *P* = 0.75).

All activities confounded, crown feathers position was similar to cheek feathers position in a significantly higher proportion of scans than what could be expected by chance (same vs. different; 69% vs 31%, Chi2 = 7.49, *P* < 0.01). The same result was observed for the pairs crown-nape (same vs. different; 66% vs 34%, Chi2 = 5.25, *P* = 0.02) and cheek-nape (same vs. different; 78% vs 22%, Chi2 = 17.01, *P* < 0.01).

### Feather posture and blushing when interacting with the caretaker

**Fig 4:**
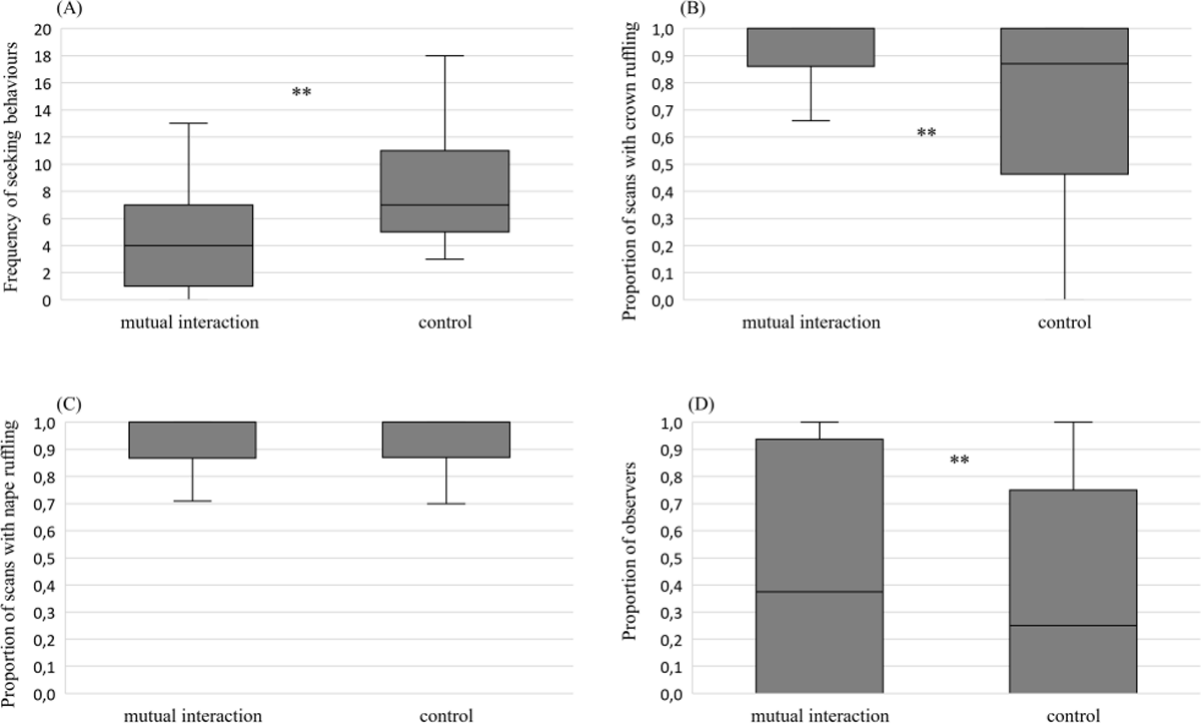
**Median and interquartile distribution ranges of number of seeking behaviours (A), the proportions of scans with crown ruffling (B), the proportions of scans with nape ruffling (C) and the proportion of observers classifying blushing as being present on photographs (D).** ** *P* ≤ 0.01 (permutation tests).

The number of seeking behaviours was significantly higher in the control condition than in the context of the mutual interaction (*df* = 1, *MS* = 420.25, *P* < 0.01) (Fig 4A). The proportion of scans with ruffled crown feathers was significantly higher during mutual interaction than during the control condition (*df* = 1, *MS* = 0.89, *P* < 0.01) (Fig 4B). The proportion of ruffled nape feather scans did not differ significantly between contexts (*df* = 1, *MS* = 0.0001, *P* = 0.9) (Fig 4C). The photographs taken when mutually interacting were classified as showing blushing around the eye by a significantly higher proportion of observers than the control photographs (*df* = 1, *MS* = 0.19, *P* = 0.01) (Fig 4D).

We found no significant effects of the random factors individual and session on the fixed effect phase for all the parameters observed (number of seeking behaviours: individual effect: df = 4, *MS* = 185.7, *P =* 1; session effect: *df* = 45, *MS* = 7.11, *P* = 1; proportion of scans with ruffled crown feathers: individual effect: df = 4, *MS* = 0.96, *P =* 1; session effect: *df* = 45, *MS* = 0.05, *P* = 1; proportion of ruffled nape feather scans: individual effect: df = 4, *MS* = 0.71, *P =* 1; session effect: *df* = 45, *MS* = 0.07, *P* = 1; proportion of observers: individual effect: df = 4, *MS* = 0.0005, *P = 0*.*95*; session effect: *df* = 45, *MS* = 0.21, *P* = 0.1).

## Discussion

We provided the first descriptive study on facial feather display and bare skin colour variations in macaws. Our data show that feather ruffling varied with the activity in which the bird was engaged and that crown feathers ruffling varied significantly with the presence or absence of mutual interaction between the parrot and caretaker. Furthermore, blushing occurred significantly more frequently during mutual interaction with the caretaker than in the control context.

**Fig 5:**
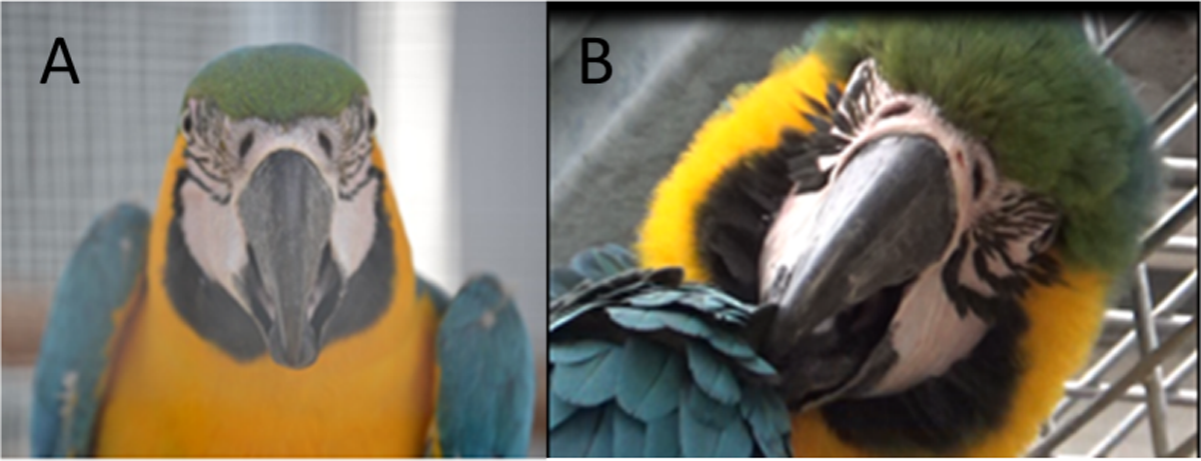
Examples of head displays. A) Photograph of a macaw with all feathers sleeked. B) The same macaw with all feathers ruffled. (All photographs taken by C. Arnould).

During the birds’ daily aviary routine, our data show a higher occurrence of feather ruffling in activities requiring no locomotion than in activities requiring locomotion. In addition, the high frequency of feathers ruffling in activities like “rest” or “chew enrichments” suggests that head feathers ruffling (crown, nape and cheek) may be associated with states of low arousal level and positive valence (e.g. relaxed, calm) [28]. Head feathers ruffling was also associated with positive social interactions and preening. In finches, ruffling the crown and nape feathers independently of the body feathers is considered a signal to concentrate mutual preening on this specific region, which is less accessible when birds preen themselves [20]. In addition, the spheroid posture engendered by ruffled body and nape feathers, is thought to stimulate clumping in social groups and to trigger the same body posturing in approaching birds [20,29]. In macaws, when ruffled, head feathers may contribute to a more spheroid posture of the head (cf. Fig 5). So far, the receiver responses to these visual displays have not been explored in avian species. In spice finches (*Lonchura punctulata*), crown-ruffling is involved in agonistic interactions [29]. In crested birds, crest rising is well described as being involved in body threat displays or sexual displays (e.g. [21,30,31]); however, in our study, no agonistic interactions were observed between parrots, and birds were non-breeding juveniles (no expression of sexual displays). The high proportion of scans with nape ruffling during low arousal levels is also consistent with observations in passerine birds. When at rest, body and nape ruffling is thought to compensate for heat loss and lower heat production [20].

In the second part of the study, we found significantly more seeking behaviours towards the caretaker during the control phase than during the mutual interaction phase. As predicted, this result showed that the birds were anticipating re-establishing mutual interaction with their caretaker highlighting a positive mutual interaction for the bird. Not yet studied in parrots, visual attention is fundamental in developing affiliative behaviour in human children [32]. Mutual attention also induces positive subjective feelings in dogs [33]. In parrots, some studies suggest that mutual attention with a trainer may be rewarding. When competing for a human’s attention, African grey parrots (*Psittacus erithacus*) change their behaviour to get the trainer’s attention [34]. Joint avian-human attention also improved learning allospecific speech [27].

Crown ruffling occurred significantly more during mutual interaction than during the control phase whereas no significant difference was found for nape feathers ruffling. Macaws may use crown and nape ruffling to solicit preening from the caretaker, but contrary to what we observed during intraspecific interaction, cheek ruffling was not expressed with the caretaker. This result indicates subtle differences in feather displays based on whether birds are engaged in intraspecific or allospecific interactions. Additional observations would be required to deepen our understanding of the crown ruffling function, but our data suggest that crown ruffling variations may be due to variations in the emotional valence between the two phases.

Blushing was identified in more photographs in the context of mutual attention than in the control context, which may also indicate different emotional valences between the two phases. In humans, blushing is due to vasodilatation of facial veins, which occurs in emotionally charged situations in the context of social attention [35–37]. Considered exclusive to humans [38], rapid colour change based on haemoglobin has been unexplored in birds despite reports on several other taxa [22]. As we did not counterbalance the session order, we cannot exclude the possibility that the birds were more aroused or stressed following retrieval from their aviary; however, more active seeking behaviours were observed (potentially higher arousal) during the control phase than during mutual interaction. This increased seeking behaviour may also indicate frustration from the bird. Whatever the cause or the emotional valence of the situation, our results show, for the first time, variation in skin colour by social context in macaws. This rapid colour change may convey subtle visual information in a social group or during allospecific interactions. Unfortunately, due to large variations in natural light in the aviaries, we were unable to analyse blushing when birds were in their social group. Additional observations are required to understand the function of blushing.

Blue-and-yellow macaws evolved to have a particularity complex face with mobile coloured feathers (black, green, blue, yellow) and bare skin. This complexity parallels the complexity found in primates. In non-human primates, complex facial coloration and bare skin colour contain social information regarding individuals’ identity, or socio-sexual status [39]. Whether primates show and detect short-term skin colour changes such as human emotional blushing remain undetermined [40]. Interestingly, facial blushing is not reported in sympatric species of blue-and-yellow macaws like the red-and-green macaws *(Ara chloropterus)* [41]. Macaws may thus provide a suitable model to investigate the social or ecological selective pressures that may have driven the evolution of facial blushing. This promising avenue of research may advance our understanding of mechanisms and function of animal coloration which is a key component of visual communication systems [42].

In conclusion, although caution must be exercised when interpreting these data due to the small sample size, we argue that crown ruffling and skin colour variation may provide facial indicators of birds’ inner subjective feelings. They may provide indicators of arousal level and/or emotional valence, two key components of animal affective states [28]. Additional investigations would help to better understand this communication system. Particularly, analysing the potential responses of receivers to visual displays is now required to determine whether birds do use variations in facial display as visual signals. We also hope to see future investigations in natural populations since captivity and human interactions may alter the way birds use facial displays. From an applied point of view, understanding visual communication in parrots may help to assess their well-being in captive conditions. Parrots are popular companion animals, with millions of parrots being kept as pets, but they are particularly vulnerable to being stereotyped [43,44]. Facial expression of emotion is well described in several mammalian species (e.g. [17,45,46]) but not in avian species. How birds use facial displays and whether they communicate their inner subjective feelings is a question that is crucial to deepening our understanding of bird sentience.

## References

[1] Andrew RJ.The displays given by passerines in courtship and reproductive fighting: a review. Ibis (1961) 103: 315–348.

[2] Lange H, Leimar O.The function of threat display in wintering great tits. Anim Behav (2003) 65: 573–584.

[3] Hill GE.Plumage coloration is a sexually selected indicator of male quality. Nature (1991) 350: 337.

[4] Griffith SC, Parker TH, Olson VA.Melanin-versus carotenoid-based sexual signals: is the difference really so black and red? Anim Behav (2006) 71: 749–763.

[5] van Dongen WFD, Mulder RA.Relative importance of multiple plumage ornaments as status signals in golden whistlers (Pachycephala pectoralis). Behav Ecol Sociobiol (2007) 62: 77–86.

[6] McGraw KJ.Melanins, metals, and mate quality. Oikos (2003): 402–406.

[7] Berg ML, Bennett ATD.The evolution of plumage colouration in parrots: a review. Emu (2010) 110: 10–20.

[8] Heinsohn R, Legge S, Endler JA.Extreme reversed sexual dichromatism in a bird without sex role reversal. Science (2005) 309: 617–619. 10.1126/science.1112774 16040708

[9] McGraw K, Nogare M.Carotenoid pigments and the selectivity of psittacofulvin-based coloration systems in parrots. Comp Biochem Physiol B Biochem Mol Biol (2004) 138: 229–233. 10.1016/j.cbpc.2004.03.011 15253871

[10] Masello JF, Quillfeldt P.Body size, body condition and ornamental feathers of Burrowing Parrots: variation between years and sexes, assortative mating and influences on breeding success. Emu (2003) 103: 149–161.

[11] Serpell JA.Factors influencing fighting and threat in the parrot genus Trichoglossus. Anim Behav (1982) 30: 1244–1251.

[12] Renton K.Agonistic interactions of nesting and nonbreeding macaws. The Condor (2004) 106: 354–362.

[13] Diogo R, Abdala V, Lonergan N, Wood B.From fish to modern humans–comparative anatomy, homologies and evolution of the head and neck musculature. J Anat (2008) 213: 391–424. 10.1111/j.1469-7580.2008.00953.x 18657257 PMC2644766

[14] Homberger DG, de Silva KN.The role of mechanical forces on the patterning of the avian feather-bearing skin: a biomechanical analysis of the integumentary musculature in birds. J Exp Zool B Mol Dev Evol (2003) 298: 123–139. 10.1002/jez.b.30 12949773

[15] Kaplan G (2015) Bird minds: cognition and behaviour of Australian native birds: CSIRO PUBLISHING.

[16] Waller BM, Micheletta J.Facial expression in nonhuman animals. Emot Rev (2013) 5: 54–59.

[17] Parr LA, Waller BM.Understanding chimpanzee facial expression: insights into the evolution of communication. Soc Cogn Affect Neurosci (2006) 1: 221–228. 10.1093/scan/nsl031 18985109 PMC2555422

[18] Scheider L, Waller BM, Ona L, Burrows AM, Liebal K.Social use of facial expressions in hylobatids. PLoS One (2016) 11: e0151733 10.1371/journal.pone.0151733 26978660 PMC4792372

[19] Olkowicz S, Kocourek M, Lucan RK, Portes M, Fitch WT, et al Birds have primate-like numbers of neurons in the forebrain. Proc Natl Acad Sci USA (2016) 113: 7255–7260. 10.1073/pnas.1517131113 27298365 PMC4932926

[20] Morris D.The Feather Postures of Birds and the Problem of the Origin of Social Signals. Behaviour (1956) 9: 75–113.

[21] Goodwin D.Further observations on the behaviour of the Jay Garrulus Glandarius. Ibis (2008) 98: 186–219.

[22] Negro JJ, Sarasola JH, Farinas F, Zorrilla I.Function and occurrence of facial flushing in birds. Comp Biochem Physiol A Mol Integr Physiol (2006) 143: 78–84. 10.1016/j.cbpa.2005.10.028 16337158

[23] Bamford AJ, Monadjem A, Hardy ICW.Associations of Avian Facial Flushing and Skin Colouration with Agonistic Interaction Outcomes. Ethology (2010) 116: 1163–1170.

[24] Ingels J, Tascon J, Giraud-Audine M.Ever seen an ‘ashen-faced’ or a ‘blushing’ Crested Caracara Caracara cheriway? Neotropical Birding (2011): 71–73.

[25] Brown L, Amadon D (1968) Eagles, hawks and falcons of the world. New York, U.S.A.

[26] Anderson PK.Social dimensions of the human–avian bond: parrots and their persons. Anthrozoös (2014) 27: 371–387.

[27] Pepperberg IM, McLaughlin MA.Effect of avian–human joint attention in allospecific vocal learning by grey parrots (*Psittacus erithacus*). J Comp Psychol (1996) 110: 286–297. 8858848 10.1037/0735-7036.110.3.286

[28] Mendl M, Burman OH, Paul ES.An integrative and functional framework for the study of animal emotion and mood. Proc R Soc Lond B Biol Sci (2010) 277: 2895–2904. 10.1098/rspb.2010.0303 PMC2982018 20685706

[29] Moynihan M, Hall MF.Hostile, sexual, and other social behaviour patterns of the Spice Finch (Lonchura Punctulata) in captivity. Behaviour (1955) 7: 33–75.

[30] Kumar A.Communication value of displays and postures in Red-vented Bulbul Pycnonotus cafer (Aves: Pycnonotidae). J Threat Taxa (2010) 2: 919–929.

[31] Ruiz-Rodríguez M, Martín-Vivaldi M, Avilés JM.Multi-functional crest display in hoopoes Upupa epops. J Avian Biol (2017) 48: 1425–1431.

[32] Emery NJ.The eyes have it: the neuroethology, function and evolution of social gaze. Neurosci Biobehav Rev (2000) 24: 581–604. 10940436 10.1016/s0149-7634(00)00025-7

[33] Nagasawa M, Mitsui S, En S, Ohtani N, Ohta M, et al Social evolution. Oxytocin-gaze positive loop and the coevolution of human-dog bonds. Science (2015) 348: 333–336. 10.1126/science.1261022 25883356

[34] Pepperberg IM (2009) The Alex studies: cognitive and communicative abilities of grey parrots: Harvard University Press.

[35] de Jong PJ, Dijk C.Social effects of facial blushing: influence of context and actor versus observer perspective. Soc Personal Psychol Compass (2013) 7: 13–26.

[36] Nikolic M, Colonnesi C, de Vente W, Bogels SM.Blushing in early childhood: feeling coy or socially anxious? Emotion (2016) 16: 475–487. 10.1037/emo0000131 26641271

[37] Crozier W.The Blush: Literary and Psychological Perspectives. J Theory Soc Behav (2016) 46: 502–516.

[38] Darwin CR (1972) The Expression of Emotions in Man and Animals. London: John Murray.

[39] Rakotonirina H, Kappeler PM, Fichtel C.Evolution of facial color pattern complexity in lemurs. Sci Rep (2017) 7: 15181 10.1038/s41598-017-15393-7 29123214 PMC5680244

[40] Bradley BJ, Mundy NI.The primate palette: the evolution of primate coloration. Evol Anthropol (2008) 17: 97–111.

[41] Forshaw JM, Cooper WT (1989) Parrots of the world: Blandford London.

[42] Cuthill IC, Allen WL, Arbuckle K, Caspers B, Chaplin G, et al The biology of color. Science (2017) 357. 10.1126/science.aan0221 28774901

[43] Kinkaid HMY, Mills DS, Nichols SG, Meagher RK, Mason GJ.Feather-damaging behaviour in companion parrots: an initial analysis of potential demographic risk factors. Avian Biol Res (2013) 6: 289–296.

[44] Van Zeeland YRA, Spruit BM, Rodenburg TB, Riedstra B, Van Hierden YM, et al Feather damaging behaviour in parrots: a review with consideration of comparative aspects. Appl Anim Behav Sci (2009) 121: 75–95.

[45] Bellegarde LGA, Haskell MJ, Duvaux-Ponter C, Weiss A, Boissy A, et al Face-based perception of emotions in dairy goats. Appl Anim Behav Sci (2017) 193: 51–59.

[46] Finlayson K, Lampe JF, Hintze S, Würbel H, Melotti L.Facial indicators of positive emotions in rats. PLoS ONE (2016) 11: e0166446 10.1371/journal.pone.0166446 27902721 PMC5130214

